# Acupuncture Therapy Is More Effective Than Artificial Tears for Dry Eye Syndrome: Evidence Based on a Meta-Analysis

**DOI:** 10.1155/2015/143858

**Published:** 2015-04-16

**Authors:** Lei Yang, Zongguo Yang, Hong Yu, Hui Song

**Affiliations:** ^1^Aerospace Center Hospital, Beijing 100049, China; ^2^Shanghai Public Health Clinical Center, Fudan University, Shanghai 201508, China

## Abstract

*Background*. The efficacy of acupuncture in dry eye syndrome patients remains controversial. *Methods*. Pubmed, Ovid, Cochrane libraries, CNKI, Wanfang, and CQVIP databases were electronically searched until October 1, 2014. Outcomes including tear break-up time (BUT), Schirmer I test (SIT), and cornea fluorescein staining (CFS) were analyzed. A meta-analysis was performed using both fixed- and random-effects models based on heterogeneity across studies. *Results*. Seven studies were included in this study; 198 and 185 patients were randomly treated with acupuncture and artificial tears, respectively. The overall BUT of patients in acupuncture group was significantly longer than that of the artificial tears group after treatment (*P* < 0.00001). The SIT was significantly higher in the acupuncture group than that in the artificial tears group after treatment (*P* = 0.001). The CFS of patients in acupuncture group was significantly improved compared to that in artificial group (*P* < 0.0001). *Conclusions*. Acupuncture therapy is effective for the dry eye patients, partly better than artificial tear treatment.

## 1. Introduction

Dry eye syndrome, a worldwide health burden, is a common ocular disorder of the tear film caused by decreased tear production or increased evaporation. Dry eye is highly prevalent, approximately affecting 14% to 33% of the adult population worldwide [1]. According to previously published literature, the overall prevalence of dry eye syndrome is estimated to be 5% to 35% in various populations [2]. The disease causes significant loss of productivity at work as there is no effective therapy [3].

Currently, in many countries, the recommended therapy for dry eye syndrome is artificial tears as lubricants or supplements for tear deficiency [4]. However, relief from artificial tears lasts only 30–40 minutes, and frequent applications are necessary, so a more effective method is needed for this disease. Acupuncture treatment which has been used for thousands of years in China and has its effectiveness has been provided for some diseases. The efficacy of acupuncture for dry eye syndrome has also been explored; recent studies [5, 6] have suggested that acupuncture should be helpful for dry eye syndrome, but other studies [7, 8] failed to reach a consensus as to which acupuncture treatment is the most effective. Some systematic reviews [9–11], which include papers that used thunder-fire miraculous moxa in treatment group, concluded proved limited evidence that acupuncture has effectiveness than artificial tears. However, the total number of randomized controlled trials (RCTs), the data accuracy, and the methodological quality in these reviews were too low to draw firm conclusions [9].

According to the concerns mentioned above, to evaluate the efficacy of acupuncture versus artificial tears for dry eye syndrome, we performed a meta-analysis based on more strict inclusion and exclusion criteria for high-quality RCTs. With more accurate methodology, we also compared the overall baseline features, including BUT and SIT, before and after treatment to avoid potential biases.

## 2. Materials and Methods

### 2.1. Search Strategy

We searched Pubmed, Ovid, Cochrane libraries, CNKI, Wanfang, and CQVIP databases until October 1, 2014. The following medical subject headings were used: “dry eye syndrome,” “dry eye,” “xerophthalmia,” “keratoconjunctivitis sicca,” “Gan Yan Zheng,” “Gan Yan,” “Zhen Ci,” “Zhen Jiu,” and “acupuncture.” Electronic searches were supplemented with manual searches of reference lists of all retrieved review articles, primary studies, and abstracts from meetings to identify other studies not found in the electronic searches. Literature was searched by two authors (L. Yang and Z. Yang) independently.

### 2.2. Study Selection

Two authors independently selected trials and discussed with each other when inconsistencies were found. Articles that meet the following criteria were included: (1) regarding study types, they should be of randomized controlled trials; (2) regarding participants, they should be dry eye syndrome patients randomly divided; (3) there should be interventions, acupuncture, and artificial tears; (4) regarding outcome measures, studies that used one or more of the following measurements were eligible: BUT, SIT, CFS, and visual analogue scale (VAS); and (5) full texts should be available.

Studies with the following situations were excluded: (1) studies that included other treatments in acupuncture group or control group (e.g., thunder-fire miraculous moxa in acupuncture group); (2) participants with Sjögren's Syndrome; (3) acupuncture combined with other treatments including warming-promotion needling, moxibustion, or Chinese herbal.

### 2.3. Quality Assessment

The methodological qualities of the included RCTs were assessed according to Cochrane Collaboration's tool described in Handbook version 5.1.0 [12]. The items related to quality assessment included random sequence generation, allocation concealment, blinding of participants and personnel, blinding of outcome assessment, incomplete outcome data, selective reporting, and other biases [12]. Two authors (Z. Yang and L. Yang) assessed the quality independently and inconsistency was discussed with a third review author (H. Song) who acted as an arbiter.

### 2.4. Data Extraction

Two researchers read the full texts independently and extracted the following contents: publication data, study design, sample size, patient characteristics, treatment protocol (points used, needle reaction time, number of treatment sessions, and frequency of treatment), and outcome measures (BUT, SIT, CFS, and VAS). The authors were contacted by e-mail for additional information if data were unavailable.

### 2.5. Definitions

Tear film BUT and SIT are considered as the primary outcomes. The BUT is performed as follows: sodium fluorescein (2.5%) was applied to both eyes and the interval between the blink of eyes and the first appearance of a dry spot or disruption in the tear film was measured. If BUT is below 10 sec, it suggests at least a moderate severity of dry eyes [13–15]. The SIT is a diagnostic method to measure the basic quantity of tear secretion. After application of local anaesthesia, Schirmer test paper (Color Bar, Eagle Vision, USA) was placed in the lateral third of the lower eyelids for 5 minutes with closed eyes. If the SIT result is below 10 mm/5 min, it also suggests at least a moderate severity of dry eyes [14, 15].

As second outcomes, CFS is based on Van Bijsterveld scoring system that intensity of stain is scored in two exposed conjunctival zones (nasal and temporal) and cornea. Score of 0 to 3 is given for each zone where 0 is for no stain, +1 for separate spot, +2 for many separate spots, and +3 for confluent spots [16]. A 100 mm VAS for self-assessment of ocular discomfort was reported by participants. Ocular symptoms related to dry eye (e.g., ocular itching, foreign body sensation, burning, pain and dryness, blurred vision, sensation of photophobia, ocular redness, and sensations of tearing) were quantified and summarised in a standard 100 mm VAS scale.

### 2.6. Statistical Methods

Data were processed in accordance with the Cochrane Handbook [12]. Intervention effects were expressed as ORs and associated with 95% confidence intervals (CIs) for dichotomous data and mean differences and 95% CIs for continuous data. Subgroup continuous data of each study were combined using the following formula [17]:

(1) SD=N1N2N1+N2M12+M22−2M1M2N1−1SD12+N2−1SD22 +N1N2N1+N2M12+M22−2M1M2 ·N1+N2−1−1N1N2N1+N2M12+M22−2M1M21/2,

where SD is the standard deviation, *N* is the sample size, and *M* is the mean.

Heterogeneity across studies was informally assessed by visually inspecting forest plots and formally estimated by Cochran's *Q* test in which chi-square distribution is used to make inferences regarding the null hypothesis of homogeneity (considered significant at *P* < 0.10). A rough guide to our interpretation of *I* ^2^ was listed as follows:

- 0% to 40% shows that heterogeneity may not be important,
- 30% to 60% corresponds to moderate heterogeneity,
- 50% to 90% exhibits substantial heterogeneity,
- 75% to 100% indicates considerable heterogeneity [12, 18].

If the eligibility of some studies in the meta-analysis was uncertain because of missing information, a sensitivity analysis was performed by conducting the meta-analysis twice: in the first meta-analysis, all of the studies were included; in the second meta-analysis, only those that were definitely eligible were included. A fixed-effects model was used initially for our meta-analyses; a random-effects model was then used in the presence of heterogeneity. Description analysis was performed when quantitative data could not be pooled. Review Manager version 5.1 software was used for data analysis.

## 3. Results

### 3.1. Study and Patient Characteristics

**Figure 1:**
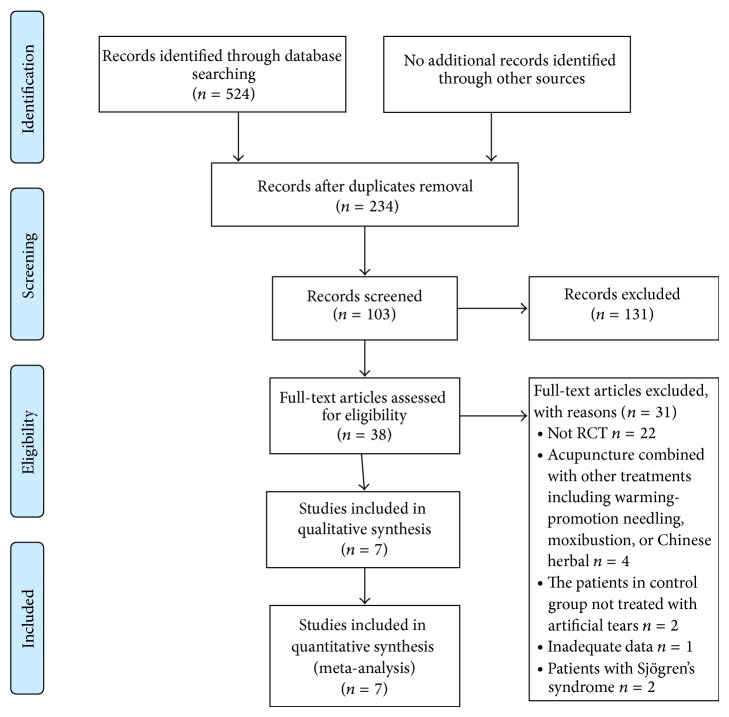
Flow diagram of the study selection process.

As shown in Figure 1, a total of 103 abstracts were reviewed; among these articles, 38 were retrieved, including 11 RCTs [5, 19–28] that are closely related to the current subject. However, three studies [19–21] were excluded because warming-promotion needling technique was used in acupuncture group and one was [22] excluded as Chinese herbal was added in treatment group. Finally, 7 trials [5, 23–28] met our inclusion criteria (Table 1). In total, 198 and 185 patients were randomly treated with acupuncture and artificial tears, respectively. The baseline characteristics of each study included in this meta-analysis are described in Table 2.

### 3.2. Methodological Quality Assessment

**Figure 2:**
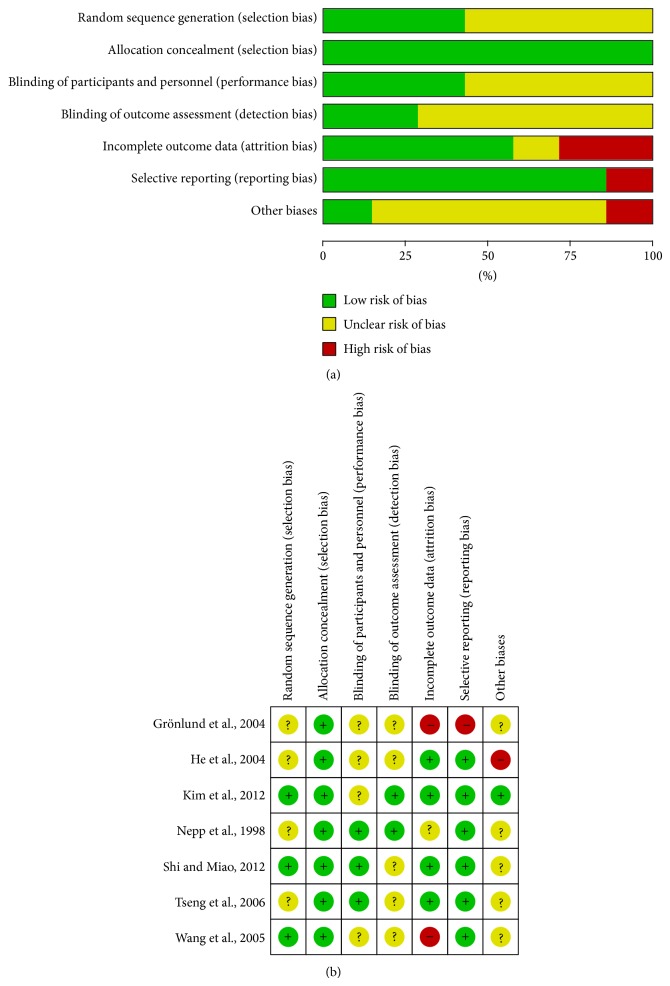
Risk of bias assessment. (a) Risk of bias graph: review authors' judgements about each risk of bias item presented as percentages across all included studies. (b) Risk of bias summary: review authors' judgements about each risk of bias item for each included study.

All studies included in this meta-analysis were described as randomized. Four studies [23–25, 27] did not report the method of randomization, but randomization was adequate in other studies [5, 26, 28]. Among these studies, two studies were randomized by a table of random numbers [5, 26] and one was randomized through computerised block-randomisation with the SAS package [28]. All studies were performed in common population, which were considered low risk in selection bias. And opaque assignment envelopes with consecutive numbers for each centre were used for allocation concealment in the study reported by Kim et al. [28]. Three reports were blind-designed [5, 23, 27]. In the study by Tseng et al. [27], apart from the acupuncturist, none of the medical professionals or technicians involved in the study knew to which group a subject had been assigned, while Shi and Miao' report [5] blinded to researchers both sample collection and examination. The outcome assessment of the study by Kim et al. [28] was analyzed by separate statistician, which was considered low risk of detection bias. More than 20% of participants were lost to follow-up in the study reported by Grönlund et al. [24] and the follow-up data was nonavailable in the study by Wang et al. [26], which were both considered high risk in attrition bias. Also, some outcomes were not reported in results section in Grönlund et al.'s study [24], which was high risk in reporting bias. Other potential biases were unclear in these trials (Figure 2). As shown in Figure 2, most of the studies included in this meta-analysis achieved low risk of biases of quality assessment items according to Cochrane Handbook for Systematic Reviews of Interventions [12]. Two studies [24, 26] had high risk of incomplete outcome data and/or selective reporting. Other biases are unclear.

### 3.3. BUT

**Figure 3:**
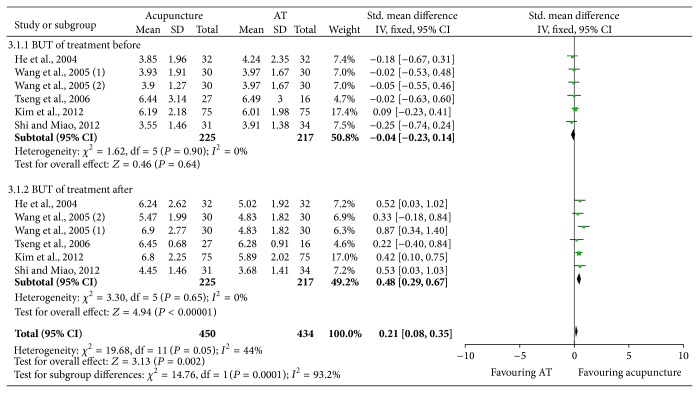
BUT comparison between acupuncture and artificial tears (AT) treatment for dry eye syndrome.

Meta-analysis of five studies [5, 25–28] demonstrated that heterogeneity was not significant among the included studies when comparing overall BUT in acupuncture and artificial tears groups both before and after treatment (*P* = 0.90, *I* ^2^ = 0% and *P* = 0.65, *I* ^2^ = 0%, resp., Figure 3). The overall pretreatment BUT of the acupuncture group and artificial tears group showed no significant difference (*P* = 0.64, Figure 3). And the overall BUT of posttreatment was significantly longer in the acupuncture group than artificial group (*P* < 0.00001, Figure 3). Similarly, the study of Nepp et al. [23] also revealed that acupuncture was more effective than artificial tears in BUT. In contrast, Grönlund et al. [24] concluded that acupuncture was no more effective than receiving a tear substitute in increasing tear quality as indicated by the BUT. Based on the results above, we assumed that acupuncture could prolong the BUT in dry eye syndrome patients.

### 3.4. SIT

**Figure 4:**
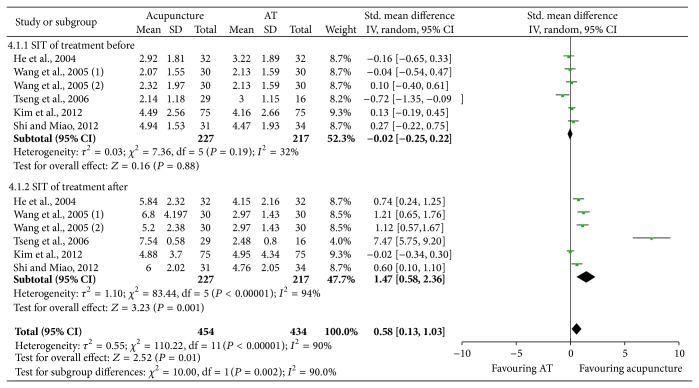
SIT comparison between acupuncture and artificial tears (AT) treatment for dry eye syndrome.

Heterogeneity was not significant among the included studies before treatment (*P* = 0.19, *I* ^2^ = 32%, Figure 4). However, the heterogeneity was significant when comparing posttreatment SIT in acupuncture and artificial tears groups (*P* < 0.00001, *I* ^2^ = 94%, Figure 4). Thus, random model of meta-analysis showed that acupuncture could significantly improve SIT compared to artificial tears (*P* = 0.001, Figure 4). Similarly, the study of Yang et al. [22] revealed that acupuncture was more effective than artificial tears in SIT. The study by Grönlund et al. [24] did not find any overall difference of SIT improvement between acupuncture and artificial tears group. However when the eyes were analyzed separately, there were a significant increase of SIT in the left eyes over time (*P* = 0.0131). Thus, patients with dry eye syndrome should beneficial from acupuncture in SIT compared to artificial tears.

### 3.5. CFS

**Figure 5:**
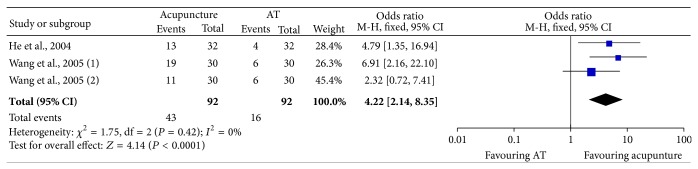
CFS comparison between acupuncture and artificial tears (AT) treatment for dry eye syndrome.

Three studies [24–26] presented CFS outcome and two [25, 26] were included in our meta-analysis. Considering that no significant heterogeneity was found among the included studies [25, 26] when comparing CFS of posttreatment between acupuncture and artificial tears (*P* = 0.42, *I* ^2^ = 0%, Figure 5), our analysis showed that the CFS of patients in acupuncture group had a significant improvement than that of artificial tears group (*P* < 0.0001, Figure 5). Since little data is available, further analysis should be performed to evaluate the efficacy of acupuncture in CFS improvement in this population.

### 3.6. VAS

Grönlund et al. [24] revealed that, according to the VAS recordings, six patients in the acupuncture treatment group felt better after finishing the treatment and no one felt worse, and in the control group no patient felt better and two patients felt worse over the same period of time (*P* = 0.036), but the positive effect had declined after completing the acupuncture treatment range of three to eight months, and there was no statistically significant difference between the two groups. In the study of Tseng et al. [27], the acupuncture group showed greater improvements in VAS values versus control group after eight-week treatment (*P* < 0.01). However, another study by Kim et al. [28] showed that statistically significant improvements did not appear at two weeks in the acupuncture group and in the artificial tears group (*P* = 0.359) or four weeks of treatment (*P* = 0.530), but significant changes were reported in the acupuncture group at eight weeks after acupuncture treatment compared with control group (*P* = 0.018). In this condition, no confirmed statement of VAS for acupuncture therapy could be presented in this meta-analysis.

### 3.7. Symptoms of Eye Discomfort

Kim et al. showed that more participants in the acupuncture group experienced general improvements of symptoms than that in the artificial tears group, but there were no significant differences (*P* = 0.06); however, a statistically significant difference was reported between the two groups in the general improvements assessed by physicians (*P* = 0.001). Other two studies [24, 27] showed that symptom of eye discomfort in the acupuncture group had improved, but no statistically significant with artificial tears group. Further researches are needed for quality-of-life evaluation in dry eye syndrome patients receiving acupuncture.

### 3.8. Adverse Event

Only two studies [24, 28] included in our meta-analysis reported adverse events. Grönlund et al. [24] reported no adverse effects of the acupuncture treatment per se were noted. In the study by Kim et al. [28], three cases of hematoma were reported in the acupuncture treatment group; 1 patient had moderate severity, and the others had mild severity. Among them, one participant declined further acupuncture treatment because of the hematoma and pain. In the other two cases, the hematomas completely disappeared in several weeks. Adverse events related to artificial tear usage were not reported.

## 4. Discussion

The therapy of dry eye traditionally involved hydrating and lubricating the ocular surface, which may provide temporary improvement in symptoms of irritation and blurred vision, but did not address the inflammation that is the underlying cause of dry eye [29]. With acupuncture being one of the oldest interventions, its mechanism in treating dry eye is unknown. Most studies [30, 31] thought that the therapeutic effect of acupuncture to dry eye syndrome was from the nervous, hormonal, and immunological systems which were closely tied to etiology of dry eye syndrome. As an active treatment for dry eye syndrome, artificial tears are generally recommended as a first-line therapy drug [32]. Considering this perspective, comparing acupuncture and artificial tears is a basic step for establishing the effectiveness of acupuncture treatment for dry eyes.

The previous meta-analyses [9–11] concluded that there were limited evidence to prove the effectiveness of acupuncture for treating dry eye due to having a large sample size and low methodological quality. Therefore, a more clear evaluation on the acupuncture and artificial tears treatments in dry eye syndromes is essential. In the present meta-analysis, we restricted our high-quality RCTs to acupuncture therapy on dry eye syndrome with non-Sjögren's Syndrome. We excluded studies that acupuncture treatment group combined with other treatments including warming-promotion needling, moxibustion, or Chinese herbal in order to reduce potential biases. In addition, we compared pre- and posttreatment indexes of the main outcomes including BUT and SIT in our analysis, which might contribute to more objective conclusions.

It is often incorrectly assumed that symptoms of dry eye are the main feature of this disease [33]. Symptom questionnaires allow for rapid and efficient collection of relevant information and can facilitate diagnosis of ocular surface disorders [34]. Questionnaires and dry eye index scores can be useful to detect the presence of dry eye and to evaluate the effect of therapeutic treatment. Three studies [24, 27, 28] of our analysis showed that the symptoms of eye discomfort had no statistically significant improvements between acupuncture group and artificial tears group. However, Kim et al. [28] showed that there were statistical significant improvements in symptoms compared to the artificial tear group. Our results indicated that high-quality trials with a large sample size are needed to estimate the efficacy of the acupuncture in improved symptoms of dry eye. Our meta-analysis suggests that the acupuncture group shows greater improvements in VAS values versus control group after treatment. The study by Kim et al. [28] also showed that there was no significant improvement at four weeks when the acupuncture group had just finished the treatment when compared to the artificial tear group, but significant changes were reported in the acupuncture group at four weeks after acupuncture treatment was compared with control group. These findings might be associated with the cumulative effect of acupuncture. Our analysis concluded that acupuncture has an effect in improving the VAS scores for patients of dry eye syndrome.

Currently available diagnostic tests and external examinations are also indispensable for every practitioner in order to reach the decision on the most suitable treatment [35]. Objective tests for dry eye can be divided into tests that examine the tears and those that examine the integrity of the ocular surface. The former can further be subdivided into tests that investigate the quantity, quality, or functional properties of tears [33].

The BUT is the most common test for determining tear film quality in use today [36]. Our results indicated that acupuncture has an effect in improving the BUT of dry eye syndrome patients. On the other hand, Schirmer I test is the most widely used technique to evaluate tear quantity [37]. Our analysis concluded that acupuncture has an effect in improving the SIT for patients of dry eye syndrome.

Cornea fluorescein staining is useful in assessing dry eye where its application can determine the integrity of the corneal and conjunctival epithelium [14, 37]. Our meta-analysis suggests that the CFS of acupuncture group had a more significant improvement than artificial group (*P* < 0.0001). Since small samples and few studies were included in this analysis, high-quality trials with a large sample size are needed to estimate the efficacy of the acupuncture in improved CFS of dry eye syndrome.

One study showed that, according to the VAS recordings [24], six patients in the acupuncture treatment group felt better after finishing the treatment and no one felt worse; in the artificial group no patient felt better and two patients felt worse at the same time (*P* = 0.036); however, no statistical significance could be found in the total number of subjective symptoms, dosage frequency, or, as indicated by the dry eye tests, tear quality, tear secretion, and ocular surface disease in the study. The author concluded that acupuncture has subjective beneficial effects in patients with dry eye syndrome.

In our analysis, we included participants with non-Sjögren's Syndrome and excluded combined treatments that might increase the positive results of acupuncture. More importantly, we compared pre- and posttreatment indexes of the main outcomes including BUT and SIT in our analysis, which might contribute to more objective conclusions. Current evidence suggests that acupuncture has superior efficacy in BUT, SIT, CFS, and VAS than artificial tears for dry eye syndrome. Acupuncture therapy is effective for the dry eye patients, partly better than artificial tear treatment. However, our review still had small sizes, relative low quality trials, and short treatment duration. High-quality randomized controlled trials with large samples, long treatment duration, double-blind design, and objective outcomes need to be conducted to evaluate the efficacy and tolerability of acupuncture in this population for a confirmed conclusion in the future. On the other hand, different acupunctulre points and methods, sustained time of acupuncture efficacy, and the correlation of acupuncture effect with treatment duration are also to be conducted.

## Figures and Tables

**Table 1:** Baseline characteristics of studies included in the meta-analysis.

Study ID	Interventions	Patients, *n*	Male, *n* (%)	Age, years	Treatment frequency	Main outcomes	Study type
Nepp et al., 1998 [23]	Acupuncture Artificial tears	30 22	NA NA	NA NA	Once per week NA	BUT, SIT, and registration of drop frequency	RCT

Grönlund et al., 2004 [24]	Acupuncture Artificial tears	12 13	1 (10) 2 (20)	61 (38–79) 51 (37–75)	Once to twice per week NA	BUT, SIT, VAS, registration of drop frequency, and questionnaire on symptoms	RCT

He et al., 2004 [25]	Acupuncture	16	NA	NA	Once in 2 days, 10 times/course, 3 courses, 10 days' rest after a course	BUT, SIT, and CFS	RCT
	Artificial tears	16	NA	NA	5 to 6 times one day, for 30 days		

Wang et al., 2005 [26]	Acupuncture 1	15	7 (46.7)	51.7 (27–75)	Once in 2 days, 10 times/course, 3 courses, 10 days' rest after a course	BUT, SIT, and CFS	RCT
	Acupuncture 2	15	5 (33.3)	51.8 (24–74)	Once in 2 days, 10 times/course, 3 courses, 10 days' rest after a course		
	Artificial tears	15	5 (33.3)	51.5 (30–73)	5 times one day, for 30 days		

Tseng et al., 2006 [27]	Acupuncture Artificial tears	17 9	6 (35.3) 6 (66.7)	47.58 ± 14.88 51.33 ± 20.91	Twice a week, 2 to 3 day apart, for 8 weeks NA	BUT, SIT, VAS, Over score of eye condition	RCT

Shi and Miao, 2012 [5]	Acupuncture Artificial tears	33 35	14 (42.4) 16 (45.7)	47.4 ± 12.70 51.4 ± 10.50	Three times a week, for three weeks Three to four times per day, for three weeks	BUT, SIT, lactoferrin concentration	RCT

Kim et al., 2012 [28]	Acupuncture Artificial tears	75 75	22 (41.5) 19 (33.9)	47.95 ± 11.11 46.05 ± 13.10	Three times per week, for four weeks at least once per day, for four weeks	BUT, SIT, VAS, Quality of life	RCT

BUT, tear break-up time; SIT, Schirmer *I* test; CFS, cornea fluoresce in staining; VAS, visual analogue scale; NA, not available.

**Table 2:** Acupuncture treatment details of studies included in the meta-analysis.

Study ID	Acupuncture rationale	Points used	Insertion depths	Responses elicited	Needle reaction time	Needle type	Number of TS
Nepp et al., 1998 [23]	NA	Local point (GB1, UB2, ST5, and Ex2YinTang), specific points for the eyes and mucosa (LI4, SI3, Li3, Kd6, and TH5)	NA	NA	30 min	NA	10

Grönlund et al., 2004 [24]	TCM	ST2, ST8, ST36, GB1, GB14, BL2, and LI4	NA	NA	30 min	NA	10

He et al., 2004 [25]	TCM	Thermal burn Yin: ST2, LI20, LI11, LI4, SP6, OR Phlegm and blood stasis mutual: ST2, SP10, SP9, SP6, ST36, and KI6.	NA	NA	20–25 min	NA	30

Wang et al., 2005 [26]	TCM	Thermal burn Yin: LI11, LI4, SP6, KI3, LI20, and ST2 Phlegm and blood stasis mutual: SP10, SP9, ST36, ST40, SP6, and ST2	NA	NA	20–25 min	NA	30

Tseng et al., 2006 [27]	NA	Ex-HN, SJ23, GB14, ST2, and SP6	NA	Gaining of qi	20 min	Number 36 one-inch needles on the face, number 32 two-inch needles for san yin jiao	16

Shi and Miao, 2012 [5]	TCM	DU20, BL1, ST1, Taiyang, SJ23, LI4, and ST36	NA	NA	25 min	40 mm × 0.25 mm,	9

Kim et al., 2012 [28]	TKM	Bilateral BL2, GB14, TE23, Ex1, ST1, GB20, LI4, LI11, and single GV23	0.6–3 cm for point at the face and head and 3-4 cm for point of hand and arm	De qi	20 min	0.20 × 30 mm,	12

TCM, traditional Chinese medicine; TS, treatment session; NA, not available.
